# The Effect of Selected Flavonoids and Lipoic Acid on Natural and Model Cell Membranes: Langmuir and Microelectrophoretic Methods

**DOI:** 10.3390/molecules28031013

**Published:** 2023-01-19

**Authors:** Paulina Laszuk, Wiesław Urbaniak, Aneta D. Petelska

**Affiliations:** 1 Faculty of Chemistry, University of Bialystok, Ciolkowskiego 1K, 15-245 Bialystok, Poland; 2 Faculty of Mechatronics, Kazimierz Wielki University, Chodkiewicz 30, 85-867 Bydgoszcz, Poland

**Keywords:** kaempferol, myricetin, lipoic acid, erythrocytes, Langmuir method, BAM methods, microelectrophoresis

## Abstract

The influence of kaempferol (K), myricetin (M) and lipoic acid (LA) on the properties of natural erythrocytes, isolated from animal blood and biological membrane models (monolayers and liposomes) made of phosphatidylcholine (PC), cholesterol (CHOL), and sphingomyelin (SM), CHOL in a ratio of 10:9, was investigated. The Langmuir method, Brewster angle microscopy (BAM) and microelectrophoresis were used. The presented results showed that modification of liposomes with kaempferol, myricetin and lipoic acid caused changes in the surface charge density and the isoelectric point value. Comparing the tested systems, several conclusions were made. (1) The isoelectric point for the DPPC:Chol:M (~2.2) had lower pH values compared to lipoic acid (pH~2.5) and kaempferol (pH~2.6). (2) The isoelectric point for the SM-Chol with myricetin (~3.0) had lower pH values compared to kaempferol (pH~3.4) and lipoic acid (pH~4.7). (3) The surface charge density values for the DPPC:Chol:M system in the range of pH 2–9 showed values from 0.2 to −2.5 × 10^−2^ C m^−2^. Meanwhile, for the DPPC:Chol:K and DPPC:Chol:LA systems, these values were higher at pH~2 (0.7 × 10^−2^ C m^−2^ and 0.8 × 10^−2^ C m^−2^) and lower at pH~9 (−2.1 × 10^−2^ C m^−2^ and −1.8 × 10^−2^ C m^−2^), respectively. (4) The surface charge density values for the SM:Chol:M system in the range of pH 2–9 showed values from 0.5 to −2.3 × 10^−2^ C m^−2^. Meanwhile, for the DPPC:Chol:K and DPPC:Chol:LA systems, these values were higher at pH~2 (0.8 × 10^−2^ C m^−2^), and lower at pH~9 (−1.0 × 10^−2^ C m^−2^ and −1.8 × 10^−2^ C m^−2^), respectively. (5) The surface charge density values for the erythrocytes with myricetin in the range of pH 2–9 showed values from 1.0 to −1.8 × 10^−2^ C m^−2^. Meanwhile, for the erythrocytes:K and erythrocytes:LA systems, these values, at pH~2, were 1.3 × 10^−2^ C m^−2^ and 0.8 × 10^−2^ C m^−2^ and, at pH~9, −1.7 × 10^−2^ C m^−2^ and −1.0 × 10^−2^ C m^−2^, respectively.

## 1. Introduction

Erythrocytes (red blood cells, RBC) are the component of blood that ensures immediate transport at the port. Normal mammalian erythrocytes take the shape of a biconcave disk 6–9 µm in size [1,2].

Mature erythrocytes do not have cell organelles, such as nuclei, ribosomes or mitochondria. They do not have a mechanism to repair damage they develop over time, and after a few months of life (approx. 120 days), they are destroyed, mainly in the spleen [3,4,5]. Their cell membranes are made of cholesterol (CHOL) and phospholipids. Choline-containing lipids, such as dipalmitoylphosphatidylcholine (DPPC) and sphingomyelin (SM), are found mainly on the outer side of the bilayer. Cholesterol molecules present in the membrane in the free form are evenly distributed in both monolayers, but they can move quickly between them [6]. The mutual proportions of the individual lipid components in both membrane layers are important for maintaining the proper shape and elasticity of red blood cells. In the membranes of normal erythrocytes, the ratio of cholesterol to phospholipids is approximately 0:9. Disturbances in the lipid composition also lead to an increase in membrane stiffness and a reduction in the ability to create multiple deformations in microcirculation [5,7]. For many years, drug-loaded red blood cells (RBC) have been exploited as delivery systems for releasing active agents into circulation, increasing life-span in the circulation of therapeutic agents, protecting through immune inactivation of therapeutic enzymes, and effecting a prolonged circulation of contrasting agents useful in diagnostic applications [8]. Research on erythrocytes has been carried out using their natural form and models. Monomolecular layers are insoluble membrane structures with amphiphilic structures. The polar part of the molecule is directed toward the water subphase, while the nonpolar component, in the form of hydrocarbon chains, is directed toward the gas phase [9,10,11]. The applied model allowed us to study the interactions between the membrane components and the factors modifying their properties. For this purpose, the Langmuir method, with Brewster angle microscopy, was used. The recorded dependence of the surface pressure (π), as a function of the surface area occupied by a single molecule in the monolayer (A) (the π–A isotherm), provided information on the degree of packing of the molecules and the phase states formed during monolayer compression [12,13,14].

In biological research, liposomes are used as model cell membranes, either diagnostic or therapeutic [15,16,17]. Liposomes are spherical structures made of one or more layers of the lipid bilayer, in which a specific volume, e.g., of water, is enclosed. The chemical modifications of lipid molecules that build liposomes increase their specificity for target ligands [18]. Liposomes, as model systems, were investigated using the microelectrophoresis technique, measuring changes in surface charge density vs. pH of the electrolyte solution [10,13,17].

The measurements were carried out using model cell membranes consisting of mixtures reflecting the composition of the outer cell membranes of erythrocytes [19]. The proportion of membrane components greatly influenced the function and properties of the RBC. In the membranes of normal erythrocytes, the ratio of cholesterol to DPPC was approximately 0.9. The research used a standard model of the outer leaflet of the erythrocyte membrane composed of DPPC and cholesterol, as well as sphingomyelin and CHOL, in a molar ratio of 10:9 [20].

**Figure 1:**
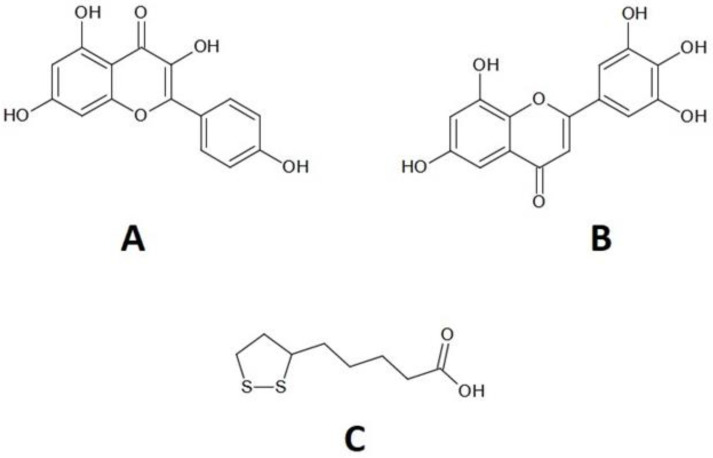
Structure of: (**A**) kaempferol (K), (**B**) myricetin (M), (**C**) lipoic acid (LA).

Kaempferol and myricetin (Figure 1A,B) are representatives of the compounds of the flavonoid group. These substances are known for their antibacterial, anti-inflammatory, and anticancer properties [21,22]. has been experimentally and theoretically studied for its ability to scavenge potentially highly harmful hydroxyl and superoxide anion radicals [23]. Kaempferol can help by increasing the body’s antioxidant defense against free radicals, which promote cancer development [24]. In addition, kaempferol is much less toxic to normal cells than standard chemotherapy drugs [25]. 

In vitro screening of fourteen flavonoids for their anticancer properties showed that myricetin was the most potent inhibitor of these flavonols. The studies conducted in the myricetin case confirmed a relationship between its structure and function and the position of the OH group in the ring [26]. Studies have shown that this compound significantly reduces the risk of pancreatic cancer [27]. A beneficial biological effect is its neuroprotective effect, giving preclinical effects in the case of Alzheimer’s, Parkinson’s, Huntington’s diseases and even amyotrophic lateral sclerosis [26,28,29]. The research was conducted with the medicinal herb Ampelopsis grossedentata. It has been observed that isolated flavonoids, such as dihydromyricetin, isodihydromyricetin and myricetin, can strongly inhibit SARS-CoV-2 3CL^pro^. The main components, or flavonoid-rich fractions, of this herbal extract can strongly inhibit SARS-CoV-2 3CL^pro^ in a dose- and time-dependent manner. It has been confirmed that strong inhibition or dysfunction of 3CL^pro^ can effectively block SARS-CoV-2 replication and further generate benefits in the treatment of COVID-19 [30].

A lipoic acid is a separate group (Figure 1C). LA is an organosulfur compound produced by plants, animals and humans that exists in nature. It has one chiral center and an asymmetric carbon, which gives two optical isomers: R- and S-lipoic acid [31]. Although the human body synthesizes LA in small amounts, the amounts of LA produced are insufficient to meet cellular energy needs. Therefore, it is obtained mainly from the diet, especially meat and vegetables. Another source of this acid is fruits [32]. LA is useful in treating several disease states where oxidative damage is believed to be very important, such as neurodegeneration, diabetes and radiation damage [33].

The examination aimed to investigate the effects of selected flavonoids and lipoic acid on the physicochemical and electrical properties of natural and model cell membranes using microelectrophoresis and the Langmuir method. Although numerous studies have used the tested flavonoids and lipoic acid, our studies were designed in this way because, to our knowledge, there are few data on the physicochemical and electrical properties of natural mono- and bilayer lipid membranes modified with kaempferol and myricetin or lipoic acid.

The cell membrane is a very important and fascinating object of research. There is no doubt that what is happening on its surface transfers into the functioning of the whole cell—and hence, the whole organism. Since living cell research is complicated, models have been used for research for many years. There has been a corresponding increase in interest in substances of plant origin, which have a healing effect on many diseases.

All functions of membranes are connected with the membrane’s structure and chemical composition. Understanding of the physiological parts of membranes helps in the understanding of the physical rights which rule their organization, because the structure and functions of membranes are closely related to each other. Therefore, the influence of selected components, e.g., kaempferol and myricetin or lipoic acid, on the electrical and physicochemical properties of lipid membranes is also of interest. Their effects will lead to a better understanding of the function of the cellular membrane and equilibria, as well as the processes in which they participate. Built-in compounds, although they do not form lipid bilayers alone, easily build in bilayers. As such, they can influence the physicochemical properties (e.g., specific area) and electric properties (e.g., surface charge density) of cellular membranes, modifying the ability of membranes to transport substances and information through the cell.

## 2. Results and Discussion

### 2.1. Monolayers

**Figure 2:**
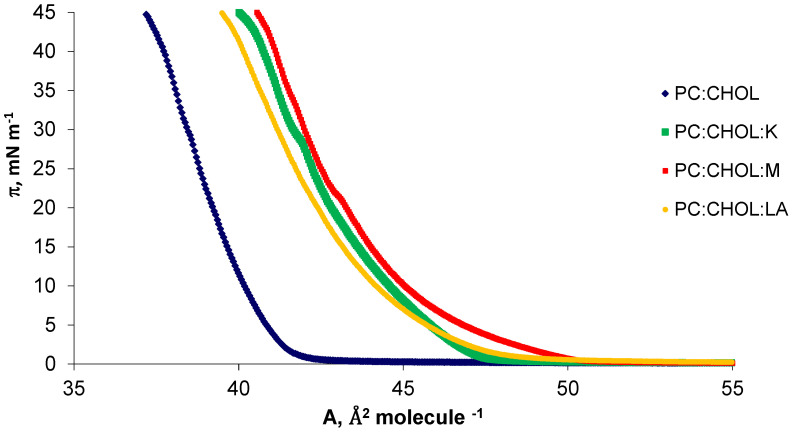
Surface pressure-area (π–A) isotherms of DPPC:CHOL, DPPC:CHOL:K, DPPC:CHOL:M, DPPC:CHOL:LA systems at a temperature of 22 °C.

**Figure 3:**
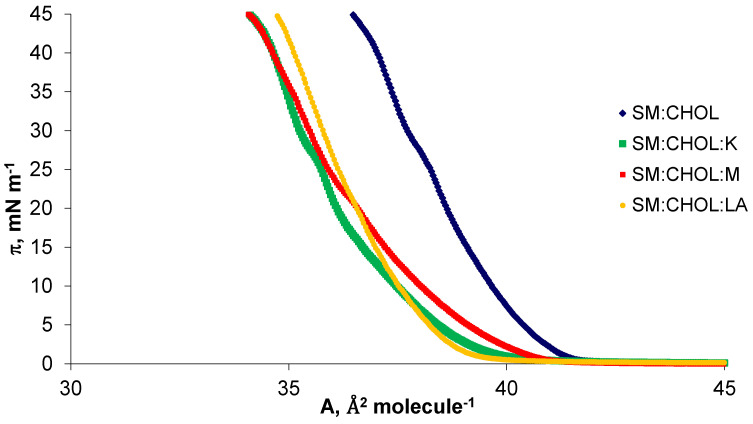
Surface pressure-area (π–A) isotherms of SM:CHOL, SM:CHOL:K, SM:CHOL:M, SM:CHOL:LA systems at a temperature of 22 °C.

The erythrocyte membrane contained phosphatidylcholine with saturated and unsaturated fatty acids. The authors modeled the erythrocyte membrane using DPPC and SM, the most frequently described and considered model phospholipids and cholesterols—the basic sterol of mammalian cell membranes. The amphiphilic structure of DPPC allowed for the straightforward and spontaneous formation of micelles, monolayers, bilayers and liposomes in contact with a polar solvent. The structure of the erythrocyte cell contained DPPC and CHOL, as well as SM and CHOL, in a ratio of 10:9 [20]. These two simple, commonly studied systems provided a good model of the erythrocyte membrane, because 80% of the entire sphingomyelin pool and 75% of the phosphatidylcholine were present in the outer layer of human erythrocytes, in contact with the environment [1]. Thus, model systems were modified with kaempferol, myricetin and lipoic acid. Using the Langmuir method with Brewster angle microscopy, the π–A isotherms were registered for pure systems and their mixtures in the tested compounds in the molar ratio 10:9:1, i.e., DPPC:CHOL:K/M/LA (Figure 2) and SM:CH:K/M/LA (Figure 3). Herein, kaempferol, myricetin and lipoic acid are denoted by the abbreviations K, M, and LA, respectively.

The surface area occupied by a single molecule in the mixed monolayer decreased with increasing surface pressure, as presented in Figure 2 and Figure 3. The π–A isotherms presented in the above figures illustrated the basic phase states: gas (G), stretched liquid (LE), and condensed (LC), with the possibility of intermediate states (G/LE, LE/LC). In the case of cholesterol, its unique feature—the condensation effect on phospholipids molecules—was noticeable. This type of surface condensation behavior is typical and widely described in the literature [34,35], in the case of mixtures of phospholipids with cholesterol. 

Studies of mixed phospholipid and cholesterol monolayers by Bañuelos-Frias et al. showed that the molecular surface of the created mixture was usually smaller than the sum of the molecular surfaces of the pure components [36]. The non-polar portion of cholesterol and the hydrophobic chains of the lipids become densely packed, as they share a limited space below the hydrophilic head groups of the phospholipids. At a specific concentration, the hydrophilic head groups of the phospholipids can no longer protect the additional cholesterol molecules from contact with water and form a cholesterol monohydrate phase [37]. In the case of the presented mixed systems with phosphatidylcholine, it was observed that these systems had a much larger specific surface area than the DPPC:CHOL mixture. The hydroxyl groups derived from flavonoids and lipoic acid can be deprotonated by forming anionic species. Therefore, complexes can form between the phospholipid and flavonoids by creating connections between the −^(+)^N(CH_3_)_3_ group of the phospholipid molecule and the −O^(−)^ group derived from flavonoids.

**Figure 4:**
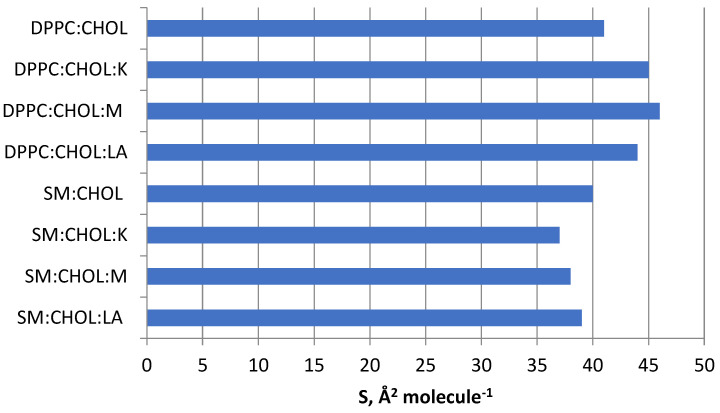
The specific surface of DPPC:CHOL, DPPC:CHOL:K, DPPC:CHOL:M, DPPC:CHOL:LA SM:CHOL, SM:CHOL:K, SM:CHOL:M, SM:CHOL:LA systems at a temperature of 22 °C.

Sphingomyelin affects the ordering of the membrane as a result of the formation of characteristic domains, together with cholesterol, called lipid rafts. They are composed of side-linked sphingolipids with a predominance of straight hydrocarbon chains, and all the free spaces between the molecules are filled with cholesterol. Strong interactions between the molecules that make up the rafts make them closely packed structures [38,39]. As shown in Figure 2, the area per molecule in SM:CHOL monolayer varied with the surface-pressure value. The specific surface area of the SM:CHOL system in a 1:1 molar ratio was reported in the literature as 37.3 A^2^ molecule^−1^, which was close to the value presented in this article [40]. As shown in Figure 3, it was observed that the SM:CHOL system had a much higher specific surface area than its mixtures with the tested compounds. The reduction of the specific surface area of the mixtures was due to the presence of hydrogen bonds between the -OH groups and strong van der Waals interactions between hydrocarbon chains. Cholesterol molecules interacted with phospholipids by arranging their polar -OH groups close to the hydrophilic groups of phospholipids. Rigid cholesterol rings interacted with hydrophobic chains of fatty acids and partially stiffened them at the point where the heads meet tails. When this occurs, lipid molecules adjacent to cholesterol have a limited ability to reposition themselves with lipids at low cholesterol sites. The greater order of the molecules stiffens the monolayer. The average area for a single molecule in a monolayer for kaempferol was 4 Å^2^ molecule^−1^ for myricetin 1 Å^2^ molecule^−1^ [10], but the lipoic acid surface was 12 Å^2^ molecule^−1^ [13]. The designated specific surfaces in the examined systems are presented in Figure 4. 

**Figure 5:**
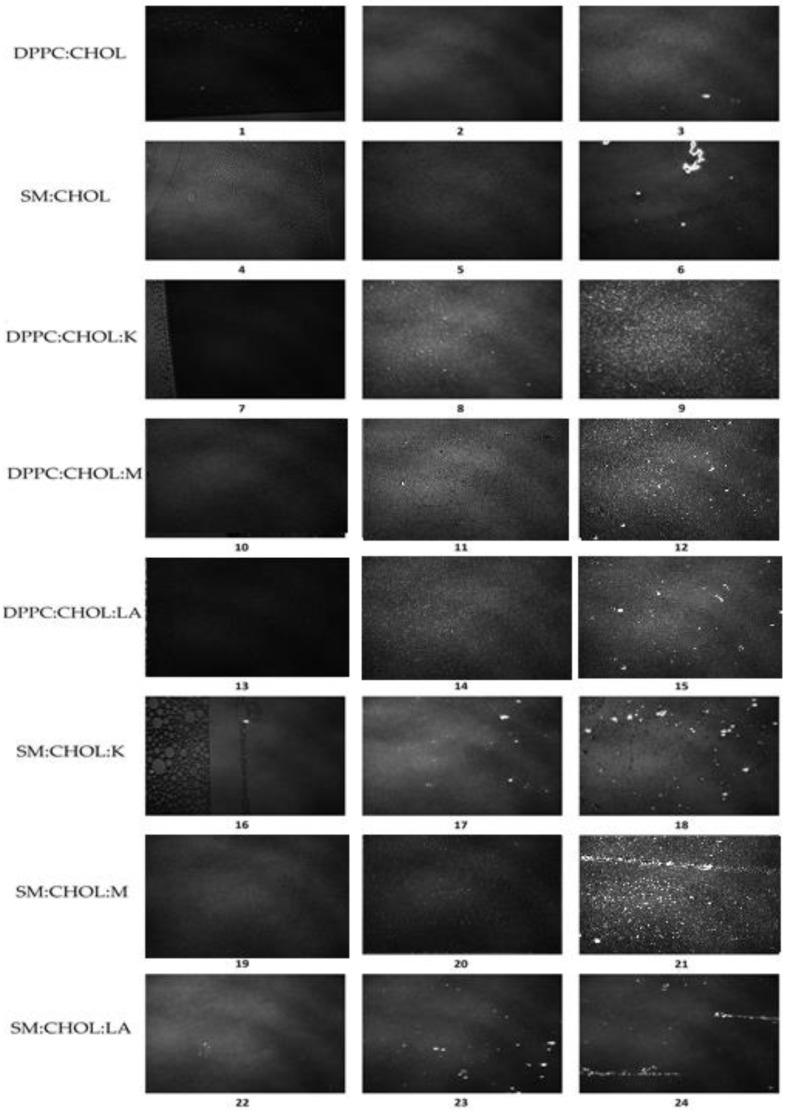
BAM images taken at a temperature of 22 °C at the following surface pressures: (**1**) 0.1 mN/m, (**2**) 29.9 mN/m, (**3**) 42.8 mN/m, (**4**) 0.1 mN/m, (**5**) 27.2 mN/m, (**6**) 40.1 mN/m, (**7**) 0.1 mN/m, (**8**) 25.5 mN/m, (**9**) 42.2 mN/m, (**10**) 0.1 mN/m, (**11**) 23.4 mN/m, (**12**) 41.2 mN/m, (**13**) 0.1 mN/m, (**14**) 23.8 mN/m, (**15**) 43.5 mN/m, (**16**) 0.1 mN/m, (**17**) 22.8 mN/m, (**18**) 44.1 mN/m, (**19**) 0.1 mN/m, (**20**) 22.4 mN/m, (**21**) 44.9 mN/m, (**22**) 0.1 mN/m, (**23**) 22.7 mN/m, (**24**) 43.9 mN/m.

During the compression of mixed monolayers at the water/air interface, phase transitions were visualized using the Brewster angle microscope (BAM) [41]. Figure 5 shows images for the tested systems DPPC:CHOL (1,2,3) and SM:CHOL (4,5,6), and their complexes with the tested compounds, i.e., DPPC:CHOL:K (7,8,9), DPPC:CHOL:M (10,11,12), DPPC:CHOL:LA (13,14,15), SM:CHOL:K (16,17,18), SM:CHOL:M (19,20,21) and SM:CHOL:LA (22,23,24). The images were captured during compression at a temperature of 22 °C and constant surface pressure in the field of view 3.6 × 4.0 mm. A black glass plate immersed in the subphase absorbed the refracted beam. The resolution of the image was approx. 6 μm/pixel.

The observation of the occurring phase changes confirmed the existence of the following phases: gas (G), extended liquid (LE), and condensed (LC), with the possibility of intermediate states (G/LE, LE/LC) for each from the tested systems (Figure 5 (1–24)). Initially, when the pressure was low, and the surface area for a single molecule was large, we observed the presence of the G gas phase (Image 1,4,7,10,13,16,19,22). Here, the molecules were disordered. There were large distances between them, which limited their interaction. Amphophilic molecules applied to the surface of the carrier phase did not completely cover the surface because the intermolecular distances exceeded the molecular size. The tested compounds K, M and LA, in their structure, contained polar -OH groups and hydrocarbon chains that did not have to assume their most ordered conformation, but could be bent. The molecules were chaotically arranged. They only interacted with each other at the moment of collision. One could lie flat on the water surface, whereas others could be perpendicular.

The images marked with the numbers 2, 5, 8, 11, 14, 17, 20e and 23 (Figure 5) showed the presence of a liquid phase (LE/LC). This was due to the gradual organization of amphiphilic molecules (the polar head was still directed toward the water phase, and the hydrocarbon chains were significantly verticalized). The decreasing distance between the polar heads allowed for stronger interactions between them, with the formation of hydrogen bonds. The LC state was equivalent to the liquid crystal state of three-dimensional bodies. In the case of DPPC:CHOL, DPPC:CHOL:K and DPPC:CHOL:LA, the foam structures characteristic of the equilibrium between gas state and liquid expanded monolayer were visible. We observed a clear transition to the condensed liquid phase in the remaining systems’ subsequent compression, resulting in tighter packing and stronger intermolecular interactions. The resulting steeper slope of the isotherm reflected a greater change in surface pressure to a comparable slight decrease in surface area. Further compression of the maximally-packed molecules led to the transformation of the 2D structure into a 3D structure. This transition, called the collapse of the monolayer, took place at a certain pressure called the collapse pressure—π_coll_. The molecules oriented themselves at the interface perpendicularly, directing the unbound -OH groups to the water phase. On the other hand, hydrophobic chains arranged themselves parallel to each other as a result of the hydrophobic effect and the van der Waals influence. Images marked with the numbers 3, 6, 9, 12, 15, 18, 21 and 24 (Figure 5) demonstrate the coexistence of a two-dimensional monolayer (dark areas) and three-dimensional crystals under collapse pressure. The displayed image contains areas of varying brightness, determined by the surface density. These structures were distinguished by a high light reflectance and took a variety of shapes.

### 2.2. Electrical Properties of Erythrocytes and Liposomes

#### 2.2.1. Erythrocytes

The natural cell membrane is a bilayer system. The inner layer consists mainly of negatively charged phosphatidylserine molecules, while the outer shell of the bilayer is made of neutral DPPC and sphingomyelin molecules [42]. Phospholipids contain groups that take on a particular charge at a suitable pH. Microelectrophoresis was used to determine the electrostatic interactions between the membrane and molecules of other substances [43]. By measuring the electrophoretic mobility, the surface charge density of the entire cell membrane was found. Determing surface charge density is crucial in detecting malignant cell transformation, as many different factors, such as membrane composition, affect the membrane surface charge [44]. 

**Figure 6:**
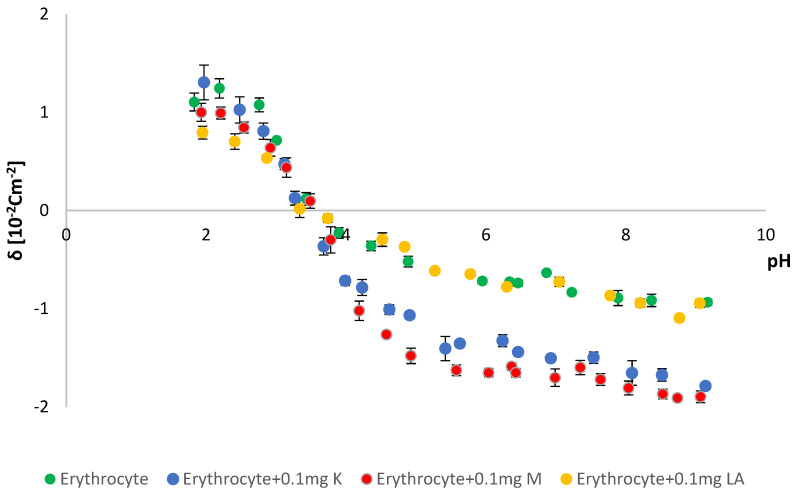
Membrane surface charge densities vs. the pH of the electrolyte solution for erythrocyte, erythrocyte + 0.1 mg K, erythrocyte + 0.1 mg M and erythrocyte + 0.1 mg LA.

To provide as much information as possible on the effects of kaempferol, myricetin and lipoic acid on natural lipid membranes, studies were carried out on natural erythrocytes isolated from animal blood. Erythrocyte solutions were prepared with the addition of 0.1 mg of tested modifying substances (K, M, LA). The research used microelectrophoresis, which indirectly enabled the determination of the electric charge on the surface of the membrane (by measuring the electrophoretic mobility) [44]. Measurements were made on the dependence of the surface charge density of the erythrocyte membrane as a function of the pH of the electrolyte (range 2–10) (Figure 6). The results are presented in Table 1.

Based on the obtained data (Figure 6), it was found that, after adding kaempferol, the value of the surface charge density decreased with the increase in the pH of the system. The addition of kaempferol to the erythrocyte solution caused an increase in the negative charge of the surface charge density in an alkaline medium (pH~9) compared to the control erythrocyte sample. In an acidic environment (pH~2), the addition of the modifying substance did not significantly affect the surface charge density of the membrane. The addition of the modifier caused a slight shift of the isoelectric point toward lower values, compared to the control sample.

After adding the modifier at pH~9, a two-fold increase in the negative charge was observed, as compared to the erythrocyte sample. As demonstrated by the data provided in Table 1, adding myricetin slightly shifted the isoelectric point value toward higher values than the control erythrocyte sample (the value changed from 3.5 to 3.6). 

Lipoic acid, as a modifying substance, was also analyzed. The above data showed that the addition of the modifying substance affected the value of the isoelectric point of a pure erythrocyte. The isoelectric point is the pH value at which a membrane’s surface charge density is 0. The electrokinetic potential, characterizing the stability of the systems, will also be equal to 0 at the isoelectric point. This means that the potential difference between the slip plane and the depth of the solution does not exist (the potential values in both areas are equal to each other). There was a slight shift of the isoelectric point toward higher values than in the reference frame. Visible changes were observed in the case of surface charge density. At pH~2, with the increase in LA content in the erythrocyte-LA mixture, the surface value of the charge density decreased compared to pure erythrocytes. However, the addition of the modifier did not affect the value of the surface charge density of the tested systems in the alkaline environment (pH~9).

Changing pH can change the charge of the hydrophilic head of phospholipids, which affects the nature of their interactions, and thus the degree of packing of molecules. Changing the pH may increase or decrease the fluidity of the film. It was observed that a change in the pH of the environment caused a change in the surface charge toward less negative values when lowering the pH (withdrawing ionization of the acid groups and increasing the degree of protonation of the amino groups), or toward more negative values when increasing the pH of the environment (i.e., reducing the protonation of the amine groups and increasing the degree of dissociation of acid groups).

#### 2.2.2. Model Membrane Systems

**Figure 7:**
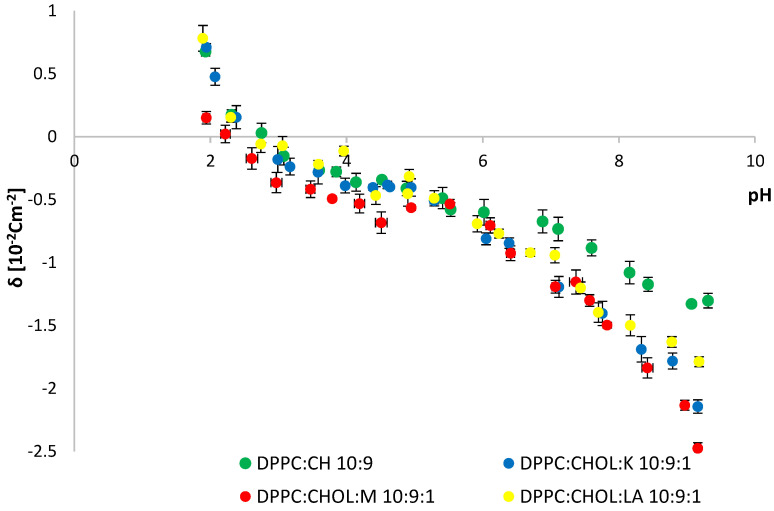
Membrane surface charge densities vs. the pH of the electrolyte solution for (DPPC:CHOL), (DPPC:CHOL:K), (DPPC:CHOL:M), (DPPC:CHOL:LA), (SM:CHOL), (SM:CHOL:K), (SM:CHOL:M), (SM:CHOL:LA) systems.

**Figure 8:**
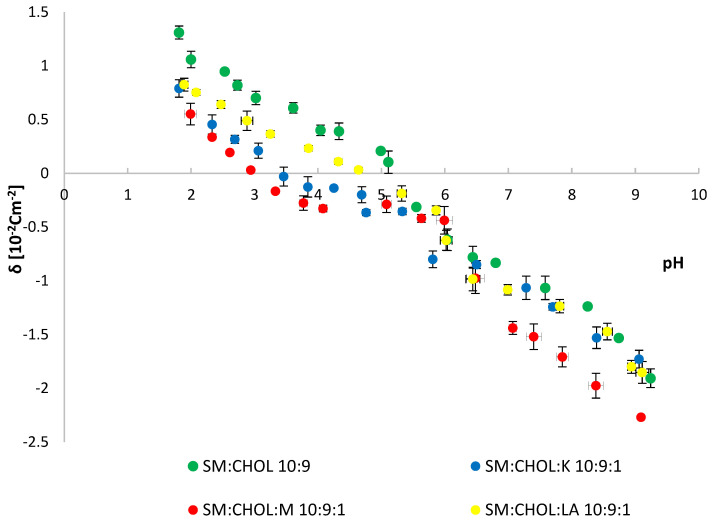
Membrane surface charge densities vs. the pH of the electrolyte solution for (SM:CHOL), (SM:CHOL:K), (SM:CHOL:M) and (SM:CHOL:LA) systems.

The structure of the erythrocyte cell, in addition to lipids, also contained sugars and proteins. Due to the complex structure of natural erythrocytes, their replacement models were introduced. Systems of DPPC and CHOL, as well as SM and CHOL, in a molar ratio of 10:9, were created. Model systems were modified with the tested compounds in a molar ratio of 10:9:1. The obtained data are presented in the charts below (Figure 7 and Figure 8) and Table 2.

The addition of the modifier to the DPPC:CHOL system shifted the isoelectric point toward lower values in each of the tested systems. The greatest change in the isoelectric point value of the pure DPPC:CHOL system was caused by the addition of myricetin. This substance significantly lowered the surface charge density of the model system at pH~2. Kaempferol did not significantly affect the value of the charge in an acidic environment. At the same time, the addition of lipoic acid slightly increased the value of the surface charge density. In the alkaline environment at pH~9, a significant shift of the surface charge density of systems, modified with the tested substances toward more negative values was observed, as compared to the DPPC:CHOL system. The strongest effect on the change in surface charge was, again, with myricetin. The addition of kaempferol and lipoic acid also shifted the surface charge density toward more negative values.

Based on the data presented in Table 2, we observed a decrease in the value of the isoelectric point of the SM:CHOL model system after adding the test substances in a molar ratio of 10:9:1. The addition of myricetin showed the greatest effect on the change in the isoelectric point, as in the case of the DPPC:CHOL system. Myricetin also caused the greatest reduction in the surface charge density of the membrane in an acidic environment and an increase in the negative charge in an alkaline environment. Kaempferol and lipoic acid showed a similar value of the membrane charge at pH~2, causing it to shift toward lower values. At pH~9, a decrease in the negative value of the membrane charge was visible with the SM:CHOL system.

The results obtained for the DPPC:CHOL and DPPC:CHOL:K/M/LA systems were compared with the results obtained for the DPPC:K/M/LA system in a molar ratio of 3:1, which were presented in previous articles [10,13]. The polar surface groups of the membrane, having an electrostatic charge, attracted the counter ions in the aqueous solution while they repelled the ions of the same sign. Thus, in the immediate vicinity of the membrane, the concentration of ions opposite to that of the net surface charge of the membrane was higher, leading to the formation of a double ionic layer. The Stern layer was formed by the adsorption of ions and polar particles at the interface. The slip plane was located in the fuzzy layer near the boundary with the rigid layer. The electrokinetic potential occurred at the boundary between bound and free liquids, between fixed ions in the adsorption layer, and free counterions of the diffusion layer. Consequently, the zeta potential was proportional to the charge density on the colloid surface, which depended on the pH. Therefore, the value of the zeta potential varied, depending on the pH and electrolyte concentration. In the case of an increase in the concentration of electrolyte ions, the thickness of the electric double layer decreased as a result of nonspecific ion adsorption, which determined the reduction of this potential value [17,44]. The addition of each of the tested modifying substances to the DPPC:CHOL system caused an increase in the negative value of the surface charge density in an alkaline environment, as compared to the control system. Similar data were obtained for the DPPC:K/M/LA systems. Modification of DPPC membranes with kaempferol, myricetin and lipoic acid caused similar changes in surface charge density at pH~9. No increase in membrane charge value at pH~2 was observed for the DPPC:CHOL system after the addition of the test substances, in contrast to the previous studies on DPPC:K/M/LA systems. The isoelectric point value for the DPPC:CHOL system after the addition of kaempferol and myricetin was shifted toward lower values, the opposite of the effect observed in research on DPPC:K/M systems. 

Monolayers and liposomes are excellent models of biological membranes. Biological membranes are not isolated systems, but are in contact with a wide variety of substances. Therefore, the results of this research could aid in achieving a better understanding of the functioning and processes taking place on the surface of membranes. Quantification of the interactions between hydrophobic surfaces and water is still a challenge for scientists. However, the data presented herein could provide valuable information on the interaction of the test substances with biological membranes. The tested compounds had an effect on erythrocytes, as well as on monolayers—liposomes made of substances that reflect the composition of natural erythrocytes. The presence of compounds building natural biological membranes represents a kind of link between the artificial and biological systems, increasing the likelihood of a positive response in the body. At the same time, it could facilitate the introduction of an active substance. The change in the value of the specific surface area of the ternary monolayers indicated the interaction of the modifiers with the cell membrane models, which were SM:CHOL and DPPC:CHOL. It was confirmed that the presence of hydroxyl groups in the molecules of flavonoids and lipoic acids caused the occurrence of hydrogen bonds—and thus, changed the physicochemical properties of monolayers and liposomes. 

The surface charge density is a very important parameter; it controls the correctness of the processes taking place in natural membranes. Surface charge plays an important role in understanding the mechanisms of membrane–environment interactions and drug delivery and is highly dependent on pH [45,46,47,48] and membrane composition [49,50,51]. The results showed that the natural compounds introduced into the membrane interacted differently if the lipid used in the research was sphingomyelin as opposed to phosphatidylcholine. Liposomes are the most commonly used carriers for the delivery of drugs or biologically active compounds, and phosphatidylcholine is the lipid most often used in research in which liposomes are made. The addition of a modifier (e.g., K, M, or LA) changed the value of the surface charge density at high pH values. Similar results were obtained for the DPPC:CHOL system modified with the tested substances; a clear decrease in the value of the surface charge density at pH~9 was observed. The results obtained for the SM:CHOL system indicated a change in the surface charge density, also at low pH values, which was not covered by natural systems. The conducted research allowed us to conclude that the PC:CHOL system was a better system and, thus, more widely studied. Although the research presented in this work was preliminary, it can be hoped that the description and interaction of the tested biologically active substances with natural membranes and their models will contribute to their use as therapeutically active substances in the future. Of course, further research is needed in order to aid in the process of designing new drugs.

## 3. Materials and Methods

### 3.1. Materials 

*Isolation of erythrocytes from whole blood:* The obtained animal blood sample (*Bost* sp. J. Meat plant. Jakim B.S.), with a volume of 2 mL, was subjected to centrifugation using the MPW-350 centrifuge for 7 min at a speed of 900 rpm. The plasma was removed, and the remaining red cell pellet was washed with saline solution (0.9% NaCl) at 3000 rpm for 15 min. The procedure was repeated 3 times.

*Membrane-forming materials:* The lipid sources were PC (≥99%) and egg yolk SM (≥95%, Sigma-Aldrich, St. Louis, MO, USA). Cholesterol (≥99%) was purchased from FLUKA. Lipoic acid (99%) was purchased from Sigma-Aldrich (St. Louis, MO, USA), while kaempferol (≥98%) and myricetin (97–103%) were supplied by POL-AURA. All reagents were used without further purification. The molecular weights of the film-forming materials used were approximately 286.24, 318.24, 206.33, 734.04, 816.20 and 386.65 g mol^−1^ for kaempferol, myricetin, lipoic acid, DPPC, SM and CHOL, respectively.

*Electrolyte solutions:* The electrolyte solutions for microelectrophoretic studies (0.155 M NaCl) and monolayer studies (pure water) were prepared using ultrapure water. The water was obtained by the Milli-Q plus water purification system with a resistivity of 18.2 MΩ cm from Millipore, Burlington, MA, USA.

#### 3.1.1. Erythrocytes Isolation 

Erythrocytes were isolated by centrifugation with 2 mL of whole blood for 8 min at room temperature at 900× *g*. The upper layer (thrombocyte-rich plasma supernatant) was removed, and the erythrocytes obtained in the lower layer were washed three times with 0.9% NaCl and centrifuged for 15 min at 3000× *g*. After the last centrifugation, the isolated erythrocytes were resuspended in 0.9% NaCl for measurements.

#### 3.1.2. Liposomes Preparation

*Liposome-forming solutions:* Chloroform (anhydrous, ≥98.5%, Avantor Performance Materials Poland S.A., Gliwice, Poland) was used as a solvent for lipids and biologically active compounds. Then, to obtain a dry residue, the chloroform was evaporated in a stream of argon. An electrolyte solution (0.155 M NaCl) was added to the obtained residue containing the tested mixtures.

*Preparation of liposomes:* Solutions of test substances with a given concentration were prepared and combined in a molar ratio of 10:9 and 10:9:1. The concentration of the tested solutions was 10 mg cm^−3^ in chloroform. Solutions were subsequently evaporated in an argon environment. Then, 5 mL of 0.9% NaCl was added to the resulting residue. Using an ice bath, the mixtures were sonicated (5 times, 1.5 min each) with the ultrasonic disintegrator UD-20 (Techpan, Puławy, Poland). In this way, liposomes containing lipids and biologically active substances were prepared.

#### 3.1.3. Monolayer Preparation—Spreading Solvent and Subphase

Chloroform ((≥98.5%), obtained from Avantor Performance Materials, Poland S.A.) was used as a solvent to prepare the solutions. (Gliwice, Poland). Solutions with concentrations of 1 mg cm^−3^ were used.

For the preparation of the kaempferol or myricetin solutions, the substance was first dissolved in a small amount of ethanol (≥99.8%) from Avantor Performance Materials Poland S.A., Gliwice, and then chloroform was added. The preparation of the lipoic acid solution did not require the addition of ethanol. Ultrapure water was used as a subphase. After each measurement, the Langmuir bath was cleaned, and high-purity methanol ((≥99.8%) from Avantor Performance Materials Poland S.A., Gliwice, Poland) was used to clean it.

### 3.2. Langmuir Monolayer Measurements

The KSV NIMA BAM apparatus from Biolin Scientific (Helsinki, Finland) was used to measure the monolayers. The apparatus consisted of a large Langmuir tub (KN 1006) connected to a Brewster angle microscope (ultraBAM nanofilm from Accurion, Goettingen, Germany) andKSV NIMA microscopy trough. The monolayers were prepared by applying the calculated volume of the substance in chloroform to the surface of the subphase using a microsyringe. Then, after 15 min (the time necessary for evaporation of the solvent), measurement commenced. The forming monolayer was subjected to continuous compression using two barriers to obtain π–A isotherms. A detailed description and the principles of operation of the apparatus were presented in the previous paper [10].

### 3.3. Microelectrophoretic Measurements

The Zetasizer Nano ZS apparatus (Malvern Instruments, Malvern, UK) was used to perform microelectrophoretic measurements. A laboratory meter WTW InoLab pH 720 (WTW, Weinheim, Germany) was used to measure pH. Measurements of electrophoretic mobility in pH 2–9 were made. Six measurements were made for each pH value of the analyzed samples (each covering 100–200 series, duration 5 s). All experiments were repeated three times. The summarized results are expressed as mean values with the determined standard deviation. The results were analyzed using standard statistical analysis, and the error bars are shown in Figure 6, Figure 7 and Figure 8.

Using the experimental data of electrophoretic mobility, the value of the surface density was determined using the Equation (1):

(1) $$\delta =\frac{\eta \cdot u}{d}$$

where η is the viscosity of the solution, u is the electrophoretic mobility and d is the thickness of the diffuse layer.

Using Equation (2), the thickness of the diffusion layer was determined:

(2) $$d=\sqrt{\frac{{\varepsilon \varepsilon }^{0}\mathrm{RT}}{2F^{2}I}}$$

where F is the Faraday number, εε^0^ is the permeability of the electric medium, T is the temperature, I is the ionic strength of 0.9% NaCl and R is the gas constant [10].

## 4. Conclusions

The research results presented in the paper concerned physicochemical properties, and included electric phospholipids modified with selected flavonoids (kaempferol and myricetin) and lipoic acid. A change in the specific surface area of the tested systems was observed in the case of mixed monolayers, in comparison with single-component monolayers. The surface charge densities of erythrocytes depended on the substance modifying its structure and the pH of the electrolyte solution. A slight shift of the isoelectric point was also observed.

Biochemical processes occurring in membranes affect the functioning of natural membranes. The functions controlled in the body can be significantly changed by various factors (e.g., poisoning, diseases or neoplastic transformation). Even small changes in the functioning of the cell affect the functioning of the cell membrane, and these changes affect the physicochemical or electrical properties of the membranes. Understanding the mechanisms underlying the interactions of the studied flavonoids with natural and model membranes is a complex problem requiring significant research.

Due to the complexity of the structure of cell membranes, the use of artificial models instead of natural systems was deemed appropriate for research. In the above work, the DPPC:CHOL and SM:CHOL systems, commonly used in research on artificial membranes imitating the erythrocyte membrane, were used [20,52,53,54].

This paper’s results showed that as little as 0.1 mg of the compound caused physicochemical changes in membranes, such as changes in the surface charge density. As there were no exact data on the actual amount of tested compounds penetrating the membrane in the literature, this research has presented an original contribution to the the body of knowledge of biochemistry and biophysics of lipid membranes. Perhaps, even a smaller amount of the compound will allow for visible changes, prompting further research. Such research studies open pathways to use the obtained results in various scientific fields. The description of the phenomena occurring in the studied systems could help us to understand the effects of the analyzed biologically active substances on pathologically-changed cells, and to develop effective methods of transporting drugs to the places where they are necessary.

## Figures and Tables

**Table 1:** The surface charge densities and isoelectric points for the erythrocyte and erythrocyte + 0.1 mg K, M, LA system.

System	Isoelectric Point	Surface Charge Density δ (10^−2^ C m^−2^)	
		Low pH Values	High pH Values
Erythrocyte	3.5	1.2 ± 0.1	−0.9 ± 0.1
Erythrocyte + 0.1 mg K	3.4	1.3 ± 0.2	−1.7 ± 0.2
Erythrocyte + 0.1 mg M	3.6	1.0 ± 0.2	−1.8 ± 0.3
Erythrocyte + 0.1 mg LA	3.4	0.8 ± 0.2	−1.0 ± 0.1

**Table 2:** Surface charge densities and isoelectric points for (DPPC:CHOL), (DPPC:CHOL:K), (DPPC:CHOL:M), (DPPC:CHOL:LA), (SM:CHOL), (SM:CHOL:K), (SM:CHOL:M) and (SM:CHOL:LA) systems.

System	Isoelectric Point	Surface Charge Density δ (10^−2^ C m^−2^)	
		Low pH Values	High pH Values
DPPC:CHOL	2.8	0.7 ± 0.2	−1.3 ± 0.2
DPPC:CHOL:K	2.6	0.7 ± 0.1	−2.1 ± 0.1
DPPC:CHOL:M	2.2	0.2 ± 0.1	−2.5 ± 0.3
DPPC:CHOL:LA	2.5	0.8 ± 0.1	−1.8 ± 0.2
SM:CHOL	5.2	1.3 ± 0.1	−1.9 ± 0.1
SM:CHOL:K	3.4	0.8 ± 0.1	−1.7 ± 0.2
SM:CHOL:M	3.0	0.5 ± 0.2	−2.3 ± 0.1
SM:CHOL:LA	4.7	0.8 ± 0.2	−1.8 ± 0.2

## Data Availability

The data presented in this study are available on request from the corresponding author.
